# Examining mindfulness-based stress reduction: Perceptions from minority older adults residing in a low-income housing facility

**DOI:** 10.1186/1472-6882-11-44

**Published:** 2011-05-31

**Authors:** Sarah L Szanton, Jennifer Wenzel, Amy B Connolly, Rachel L Piferi

**Affiliations:** 1 Department of Health Systems and Outcomes, Johns Hopkins University School of Nursing, Baltimore MD, 21205, USA; 2 The Department of Acute and Chronic Care, Johns Hopkins University School of Nursing Baltimore MD, 21205, USA; 3 Independent Contractor, Baltimore MD, 21205, USA; 4 Department of Psychological and Brain Sciences, The Johns Hopkins University, Baltimore MD, 21218, USA

**Keywords:** mindfulness, older adults, stress reduction

## Abstract

**Background:**

Mindfulness-based stress reduction (MBSR) programs are becoming increasingly common, but have not been studied in low income minority older populations. We sought to understand which parts of MBSR were most important to practicing MBSR members of this population, and to understand whether they apply their training to daily challenges.

**Methods:**

We conducted three focus groups with 13 current members of an MBSR program. Participants were African American women over the age of 60 in a low-income housing residence. We tape recorded each session and subsequently used inductive content analysis to identify primary themes.

**Results and discussion:**

Analysis of the focus group responses revealed three primary themes stress management, applying mindfulness, and the social support of the group meditation. The stressors they cited using MBSR with included growing older with physical pain, medical tests, financial strain, and having grandchildren with significant mental, physical, financial or legal hardships. We found that participants particularly used their MBSR training for coping with medical procedures, and managing both depression and anger.

**Conclusion:**

A reflective stationary intervention delivered in-residence could be an ideal mechanism to decrease stress in low-income older adult's lives and improve their health.

## Background

Programs teaching mindful awareness to promote well-being have become a recent topic of inquiry among researchers [1]. Rooted in Buddhist traditions and formally developed by Jon Kabat-Zinn in the 1980s, mindfulness has developed secularly to describe a process of becoming aware of present experience and, through that awareness, reducing emotional and physical distress [2]. Mindfulness has been described as more than merely a relaxation technique; rather, it is mental training to develop awareness and acceptance skills to cope with daily events that may otherwise lead to heightened anxiety and stress [3]. Mindfulness-based programs enhance awareness and acceptance of conscious states and teach people to approach daily situations "mindfully" [4].

Research demonstrating the efficacy of mindfulness-based stress reduction (MSBR) in promoting health is mounting although randomized, controlled studies and the inclusion of minority older adult populations are both largely absent from the literature. Past research on mindfulness-based stress reduction (MBSR) programs has demonstrated enhanced coping, improved self-efficacy, and better symptom control in general populations without control groups [3,5]. The few randomized controlled trials of MBSR have been in specific clinical populations such as cancer [6], rheumatoid arthritis patients [7]or those with chronic pain [8]. Studies have shown MBSR to be effective in treatment of chronic pain [2,8-10], anxiety disorders [11], and hypertension [12]. Reibel et al. demonstrated that MBSR increases health-related quality of life and social functioning, while decreasing physical pain, role limitations caused by physical health, and anxiety and depression among mixed diagnosis patients [13]. While MBSR programs have been shown to be related to positive outcomes, the research evidence to date hass been limited to clinical populations [3]. Further, little evidence exists for the efficacy of MBSR programs among older adults [5]or in low-income, urban minority populations [14].

Older adults, we hypothesized, could be a particularly responsive group to train in mindfulness due to their life experience accompanied by a potentially increased ability to examine their lives as well as the capacity, honed by aging-related changes, to accept their life. Low-income minority older adults, we further hypothesized, might have more stressors[15], more depression [16,17] and more social isolation [18,19] and thus more possible benefit from mindfulness training. However, there was no literature base or prior reports of experience with this population to indicate low-income minority older adults' acceptance of training that has formerly been associated with East Asian religious practices. Therefore, the purpose of the current study was to examine the perceptions of a mindfulness-based program in a sample of low-income minority older adults. Due to the mounting evidence regarding its effectiveness in reducing stress, increasing self-efficacy, and managing physical ailments, mindfulness was proposed as an effective intervention for minority older adults living with multiple social and physical concerns. Our team is currently using a randomized clinical trial to test these hypotheses. The goals of the present study were to assess older minority adult participants' openness to the intervention, understand which components of the intervention were most important to them, and examine their application of the mindful awareness training outside of the intervention. Determining acceptance or openness to this intervention was an important first step in acquiring information that could be used to develop further mindfulness-based interventions for this underserved population at high risk for chronic multi-morbidities.

In order to determine openness to this intervention, we decided to ask a group of older low-income African -American adults about their perceptions of the program. Based on their answers, we could tailor future programs. We decided on a focus group methodology to accomplish this.

Focus group research is a qualitative method of collecting data that allows researchers to understand experiences from the perspective of the participants (Patton, 2002). Focus groups allow participants to interact with each other as they answer questions posed by the researcher. In doing so, they allow for individuals to disagree or expand on other participants' comments. Focus groups are a useful approach to elicit first-hand information from participants. The rising popularity of focus groups highlights the uniqueness of their group context. This allows for the facilitation of efficient data collection and the potential to elicit participant views related to experiences involving motivations, feelings, attitudes and opinions on health-related issues among challenging and/or vulnerable target populations [20,21]. Advantages of using focus groups include: (1) being less intimidating than one-on-one interviewing but providing more depth than questionnaires (2) having sensitivity to participants' culture and age by acknowledging participants as experts and obtaining insight into participants' own language and concepts (4) allowing group interactions to take place, (3) permitting researchers to learn more about the degree of consensus on a topic, and (4) encouraging dialogue and dialectic between researchers and participants in order to mutually identify, describe, analyze and attempt to resolve key issues [22,23]. Because oral history is a strong tradition in many minority communities, inviting participants to describe their experiences is generally believed to be an effective approach to data collection with minority older adults.

## Methods

In order to collect data regarding the perceptions of a mindfulness-based program implemented among a group of low-income minority older adults, a focus group design was selected. Three focus groups were one hour each conducted with members of ELDERSHINE, a mindfulness-based program designed for older adults.

### *The *ELDERSHINE *Program*

ELDERSHINE is a psycho-educational program designed to foster awareness of internal states; promote positive mental and physical health through mindfulness; and build individual and neighborhood capacity through civic engagement and the creation of a caring community. The design of a series of eight workshops creates a sequential and cohesive program. The program teaches mindfulness-based stress reduction skills adapted from Jon Kabat-Zinn's MBSR program and was developed by one of the co-authors (A.B.C.) to teach older adults how to meditate and use mindfulness in their daily lives. Modifications from MBSR include: briefer meditation periods; shorter weekly sessions; emphasis on seated meditations rather than mindful movements or walking meditation (due to physical limitations of participants); and no daylong retreat. In lieu of a workbook, participants received a folder and weekly handouts were given out at each session with program concepts and poetry in large type to facilitate easier reading.

ELDERSHINE invites participants to discover and mobilize their strengths and foster resiliency within themselves as individuals and as a community of elders who can share wisdom and strength as they support and nurture one another. The ELDERSHINE core program consists of eight 90 minute sessions. Each weekly session includes three guided meditations; an opportunity to discuss participants' home practice and integration of mindful skills; a time for positive sharing (called sharing "Victories"); and a group process that allows participants to learn through reflection on their own experience, activity and supportive dialogue. A nourishing snack at a "tea party" served after each workshop encourages socialization, extended conversation among participants, and a focus on healthy eating.

### Participants

Participants for the study included members of an ELDERSHINE program being conducted in a low-income senior housing facility in Baltimore, Maryland. The moderator of the ELDERSHINE group verbally invited members to participate in the focus groups. The moderator also announced she would not attend the focus groups. Those interested were called the night before to increase attendance. Thirteen individuals participated in the study. Three separate small focus groups (of 4, 4, and 5 participants) were conducted to increase participation in discussion by having small groups. This is within the optimal range of 4-8 participants [23]. These groups included almost every regular ELDERSHINE attendee. All participants were low income African-American women over the age of 60 years old and younger than 90. They were all Protestants (Baptist, Methodist, and Apostolic). Their education level ranged from less than 8 years of school through obtaining some college education. This study was approved by the IRB at the Johns Hopkins School of Public Health. All participants provided oral consent.

### Procedures

Our team tape-recorded the focus group sessions, and both the moderator and the trained research assistant took notes during and after the sessions. A focus group interview guide and probes guided each focus group session. Two of the groups were moderated by one of the investigators on the project (S.L.S), and one was moderated by another investigator (R.L.P). The same trained research assistant took notes during all sessions and transcribed the sessions from the audiotapes afterwards.

For each session, the moderator started the discussion by informing the group of the purpose of the session. Any questions that the participants had were answered prior to beginning the discussion.

After all participants' questions were answered, the following four questions were asked to guide discussion:

1. What has ELDERSHINE meant to you?

2. Do you feel that you have changed in any way due to your involvement in ELDERSHINE?

3. What parts of ELDERSHINE have meant the most to you?

4. How would you describe ELDERSHINE to others?

### Analysis

Following each focus group session, the audiotapes were transcribed verbatim by the research assistant into Microsoft Word 2000. Primary themes from the focus groups were identified by inductive content analysis (Patton, 2002; Straus & Corbin, 1990). The level of analysis was across cases and across sessions to allow for themes to be developed overall for all participants.

The focus group interview guide dictated the topics of data analysis.^31 ^The investigators first analyzed the data with multiple readings of interview transcripts to gain a general sense of the data along with review of researcher field notes which included researcher observations on communication factors including body language, gestures, tones and voice intensity [23]. We meticulously recorded initial findings to maintain a clear audit trail. Preliminary coding of concepts followed, with categorization and a search for themes (see steps, below). We examined the data for themes, patterns, commonalities and variation. We continually validated categories, themes, and conclusions by referring back to the data. We also examined theme variations.

We achieved data trustworthiness through collection and review of field notes by the trained research assistant at each focus group session, as noted above. Each investigator described and interpreted their own behavior and experiences in relation to the research and each participant which enhanced the credibility of the data [24]. We used field notes to provide a clear decision trail concerning the study, describing and justifying what was actually done and the reasons for doing so [24].

Judgment regarding trustworthiness and authenticity was made using member checking and participant review techniques described by Lincoln and Guba [25]. Participants were assured that their comments would be reported in a confidential manner, using pseudonyms in the actual group sessions, in transcripts and in research reports.

## Results

Analysis of the focus group responses revealed three primary themes in the ELDERSHINE program: 1) stress management; 2) learning, practicing and using mindfulness, and 3) social support. Participants in ELDERSHINE articulated perceived benefits of the program as well as their ability to acquire and transfer skills learned in the program to their everyday lives.

### 1) Stress management

The first question that was asked in the focus group sessions was, "What has ELDERSHINE meant to you?" The primary theme that emerged across all of the focus groups was the ability to use meditation skills in coping with stressors in their life. These stressors included, growing older with some physical pain, medical tests, financial strain, and having children/grandchildren with significant mental, physical, financial or legal hardships. As an example of how ELDERSHINE participation had helped with stress management, one participant remarked:

"And then with the meditation part of it, it just takes you away from your everyday concerns in life and for that, for that moment, you are in such a restfulness. You don't even know you're really sitting there in the chair sometimes, you're somewhere, wherever, [you know] the meditation might take you. And, I think that's what I like about coming to ELDERSHINE.

Another participant stated:

"When I meditate it's like a soothing thing inside, and relaxing. Very relaxing. You don't think about, you don't actually hear nothing, you don't hear nobody. And it's, just like I said before, it's very soothing. It's better than taking a massage, put it to you that way. All the things that really [bug] and bother you at that particular time melts away and then your whole day is a good day because it doesn't, to me, actually, I don't have these pains until I maybe bend over to do something, but other than that, it don't bother you. It's just a good relaxing day. When you go to bed at night, you really can relax, you can sleep, have a very good peaceful night. And then, the next day, when you're not in ELDERSHINE, you can do the same thing then, but you might not do it at the same time, but at a different hour of the day."

Furthermore, the meditation was described as spiritual and related to God by this group. One participant remarked:

"ELDERSHINE is a meditation of our spiritual [lives]. We might not mention God's name all the time, but He's there all the time. It is our spiritual self that's being nurtured."

This distinction between spirituality and religiosity is one that is often made much of in academic circles. However, from the perspective of our Protestant participants, people with traditional religious commitments are willing to learn and practice meditation without fear of supplanting their usual religious practices.

### 2) Applying meditation to their daily lives and stresses

A second theme that emerged was that mindfulness meditation was a new practice and one which they were learning to apply to different areas of their lives. Participants revealed that they learned how to meditate and how to use meditation in their lives to reduce stress. One participant stated:

"I had to go to the hospital for an MRI, and, I told Amy [the program designer and interventionist] I took her voice with me, going into the MRI you know, I love the Lord very much, and I'm not taking away from God, but I took her with me with God into the MRI going through the tube and all the "clunk, clunk, clunk" noise you hear in there. I took Amy's voice with me to help, you know, help me, quiet me down and I wouldn't have to go through all that [without it]."

Several other participants offered examples of ways they were applying the skills they were learning

"And when you get depressed or you feeling sad, you meditate, it helps. I have experienced that the past week, you understand? And it's really helped me by meditating."

"You can always learn something and learn how to meditate. Meditat[ion] is giving yourself a chance to think before you speak. Knowing what you're doing. It slows you down, [to think] of better things."

"You catch yourself getting all upset or kind of worked up or disturbed a bit, so [you say] 'let me go on my break"... I have a special chair that I sit in now.... All the thoughts and bad feelings you had all been melted away because your body is relaxed into your meditation. And then you come out of your meditation, you really feel like a really good person. You feel more relaxed and you can go about doing what you have to do."

### 3) Social Support

An additional theme that emerged was that ELDERSHINE allowed participants to make friends and to share their lives. One participant described this theme in the following manner:

ELDERSHINE, it brings the different neighbors together and that they can talk and they can have some peaceful time together. Bringing people together and they feel comfortable talking about different things in life because they get to know the people in the circle."

In addition to sharing with each other, participants specifically mentioned sharing "victories" as an important component of ELDERSHINE. This is a time in which each participant describes something that brought them joy or that they accomplished during the week, even as simple as getting out of bed in the morning. One participant described this part of the weekly program when she stated:

"You go in every week you come in and you talk about your victories, what [you] might have done that week, something you, in your life that was just a great victory to you. So you get to share in front of everybody your victory."

The building in which ELDERSHINE takes place is a high rise for low-income older adults and has no common spaces or common programs. A final theme that emerged was that being in this group increased their social support, specifically the number of people that they believed they could turn to for assistance or for whom they could provide support. One participant commented:

"When I first came here, I didn't call nobody but my sister. My sister live on the seventh floor, When I [don't know who] I'm gonna call, I call my sister. But, now I've got plenty of people, I call them."

Another participant stated:

"You come together, you know, and know your neighbors and talk to them, understand, you know? [When they have] something going on, they've got problems too, it's really good, it's really good."

Participants reported feeling connection to and respected by others in the group. As one participant remarked,

"I would tell them, it's a place to meet friends and share thoughts with one another."

Sharing victories and talking with each other were among the ways that the participants reported that they learned respect for one another, focused on others, and fostered valuable connections and enhanced communication skills. As one participant reported,

"It teach[es] you how to respect each other, how to listen to each other and don't talk while somebody's talking."

## Discussion

In this focus group study with low-income minority older adult participants of a mindfulness-based program, we found that those who were in the intervention reported learning meditation skills, used it to cope with stressors in their lives, and increased their sense of community in a socially-isolating building.

These findings, though limited to female participants of one mindfulness program in one low-income housing project, are important. Because there is evidence that mindfulness programs may help in management of illness and low-income minority adults often suffer from multiple chronic health problems, our findings could suggest further research in an underserved population with this low-cost mindfulness-based intervention. Further, older adults are at a time in life when a reflective, stationary intervention, delivered in-residence could be an ideal mechanism to improve health. The demonstrated ability to incorporate the training into use with stressful medical tests and other challenging episodes during the course of day-to-day life shows that the benefits may reach beyond the in-class training.

Additionally, social support is important in both health promotion and during treatment for diseases like cancer, particularly among underserved populations. For example, informal and formal social support networks are believed to decrease barriers to cancer treatment in African-American older adults. In a study performed by Guidry et al, [26]African American cancer patients were more likely to report a need for formal and informal sources of support during treatment than were Caucasian Americans. Older African Americans may require enhanced and multi-faceted cancer support because they are known to possess generally poorer health status and fewer financial resources than their non-minority counterparts [27].

Future research should examine the specific psychological, social, and physiological effects of a mindfulness-based stress reduction program for older adults. This focus group study has revealed that program participants, in addition to being receptive and willing to participate in the program, enjoyed the meditation and mindfulness practices, continued to effectively utilize the techniques outside of the program, and gained social support from the program. All themes emerged in all three focus groups which encompassed the bulk of the data. There is some homogeneity of experience because of the demographic homogeneity, the common sharing of experience in the meditation group, and the fact that they all participants live in the same apartment building. Though the themes were broad, they did not warrant sub-categorization. Our team has also conducted a randomized clinical pilot trial of mindfulness meditation training with low- income minority older adults at a separate low-income housing building and with people who have never tried meditation [28].

A few limitations should be mentioned. The participants were only women. It is unknown whether men would have the same reactions to the ELDERSHINE program. However, low income minority communities of older adults are predominantly women. A second limitation is that the participants had been participating in ELDERSHINE for a range of time, some as many as three years. This is both a limitation and a strength. The limitation is that we cannot be sure these findings would apply to those who participated in the more typical 8 week mindfulness program. The strength is that, for the interviewed participants, the program is clearly relevant and sustainable demonstrated by the continued participation. A third limitation is the focus group participants knew each other. This may have limited negative comments due to social desirability bias [29]. A fourth limitation is that we did not stop the sampling due to data saturation but rather stopped due to near universality of focus group attendance by the ELDERSHINE participants. Because we offered the focus groups in the participants' apartment building at a convenient time and offered food, the three focus groups included virtually everyone in the mediation group.

## Conclusion

In conclusion, this study has shown preliminary acceptability and perceived benefits evidence of a mindfulness-based stress reduction program for low-income minority older adults. Similar to research that has shown that mindfulness-based interventions may be effective to reduce stress, increase self-efficacy, and manage psychological and physical ailments among younger populations, study findings suggest that similar benefits may be achievable in older, more vulnerable populations.

## Competing interests

The authors declare that they have no competing interests.

## Authors' contributions

SLS provided some of the focus groups, collaborated on the analysis and drafted the manuscript. JW participated in the analysis and the writing of the manuscript. ABC provided the meditation groups and participated in the writing of the manuscript. RLP provided some of the focus groups, collaborated on the analysis and participated in the writing of the manuscript. All authors read and approved the final manuscript.

## Pre-publication history

The pre-publication history for this paper can be accessed here:

http://www.biomedcentral.com/1472-6882/11/44/prepub
